# Optical Anapole Modes in Gallium Phosphide Nanodisk with Forked Slits for Electric Field Enhancement

**DOI:** 10.3390/nano11061490

**Published:** 2021-06-04

**Authors:** Jingwei Lv, He Zhang, Chao Liu, Zao Yi, Famei Wang, Haiwei Mu, Xianli Li, Tao Sun, Paul K. Chu

**Affiliations:** 1 School of Physics and Electronic Engineering, Northeast Petroleum University, Daqing 163318, China; lvjingwei2009123@126.com (J.L.); zhanghe1996123@126.com (H.Z.); wfm_lele@126.com (F.W.); yxt0407@163.com (H.M.); lxl7158@163.com (X.L.); 2 Heilongjiang Provincial Cultivate Collaborative Innovation Center for Geothermal Resources Efficient Development and Comprehensive Utilization, Daqing 163318, China; 3 Joint Laboratory for Extreme Conditions Matter Properties, Southwest University of Science and Technology, Mianyang 621010, China; 4 Media Lab, Massachusetts Institute of Technology, Cambridge, MA 02139, USA; taosun@hotmail.com.hk; 5 Department of Physics, City University of Hong Kong, Tat Chee Avenue, Kowloon, Hong Kong 999077, China; 6 Department of Materials Science and Engineering, City University of Hong Kong, Tat Chee Avenue, Kowloon, Hong Kong 999077, China; 7 Department of Biomedical Engineering, City University of Hong Kong, Tat Chee Avenue, Kowloon, Hong Kong 999077, China

**Keywords:** dielectric nanostructures, anapole modes, gallium phosphide, electric field enhancement

## Abstract

High refractive index dielectric nanostructures represent a new frontier in nanophotonics, and the unique semiconductor characteristics of dielectric systems make it possible to enhance electric fields by exploiting this fundamental physical phenomenon. In this work, the scattered radiation spectral features and field-enhanced interactions of gallium phosphide disks with forked slits at anapole modes are investigated systematically by numerical and multipole decomposition analyses. Additional enhancement of the electric field is achieved by opening the forked slits to create high-intensity hot spots inside the disk, and nearby molecules can access these hot spots directly. The results reveal a novel approach for near-field engineering such as electric field localization, nonlinear optics, and optical detection.

## 1. Introduction

Nanophotonic structures support resonant modes with subwavelength field confinement and enable strong light–matter interactions. In the past decade, metal plasmonic nanostructures have attracted a great deal of attention because of their large field enhancement and small module volume. In addition, metal plasmon nanoparticles made of different materials with various shapes [1,2] and different surface structures have been used to couple the electromagnetic energy with molecules to enhance fluorescence [3,4,5] and surface-enhanced Raman scattering (SERS) [6,7]. However, in practical applications, intrinsic absorption, external heating, and other factors inevitably impact the metals [8]. As an alternative to metallic nanostructures, dielectric nanostructures with high refractive indices and low loss are of great interest due to their ability to support electrical and magnetic resonance in the optical frequencies.

Nanoantennas, upon visible light irradiation, can compress the optical energy into a volume less than the diffraction limit [9,10,11]. This process produces a large enhancement in the local electromagnetic field to augment surface-enhanced infrared absorption (SEIRA) [12,13,14], photothermal biosensing [15], spontaneous emission [16], and nanolasing [17]. To obtain the maximum electromagnetic field enhancement factor, various approaches have been proposed, for instance, designing an ion-metal dimer structure with a small distance between two plasma-metal nanostructures for maximum amplification [18] and adding a glass substrate under the disk [19]. Optical anapoles are often realized with metallic materials with complex geometries [20,21,22]. However, high intrinsic absorption losses and associated local heating of the plasmonic nanostructures severely limit their practical use in many scenarios. In spite of recent advancements to improve the near-field strength, it is still necessary to achieve low loss, and hence, dielectric structures with high refractive indices and strength have been an effective alternative to plasmonic metal antennas.

In this work, a nanoantenna comprised of a gallium phosphide (GaP) nanodisk with a forked slit is designed, and the field enhancement effect is investigated. Compared with silicon, GaP has negligible linear absorption and a larger Kerr nonlinear index (n_2_) [23]. The contribution of the single-hole disk to the scattering cross section and electric field enhancement under plane wave irradiation is determined, and the nanoantenna with the forked slit exhibits a strong electric field due to its unique geometric parameters. By introducing a circular hole in the center of the disk, the strongest electric hot spot is exposed to the outside of the particle. Under these conditions, the maximum electric field enhancements are 43.7 at the first-order anapole mode and 55.7 at the second-order anapole mode. If the circular holes are rotated in a proper combination to form a forked slit, maximum electric field enhancements of 155.7 at the first-order anapole mode and 1129 at the second-order anapole mode are produced. The electric field enhancement increases to 1063, which is approximately 100 times that of the disk-shape nanodisk. The remarkable enhancement factor is revealed by the optical anapole model, in which far-field scattering is close to the minimum and the near-field energy is at the maximum [24].

## 2. Theory

To increase the strength of the electromagnetic field, destructive interference between the electric dipole and magnetic dipole is exploited. Precise analysis of the multimode decomposition of the scattering cross section of the nanodisk can estimate the impact on the nanodisk’s scattering. To elucidate the contribution of different modes, multipole decomposition, involving the electric dipole moment (ED), magnetic dipole moment (MD), circular dipole moment (TD), electric quadrupole moment (EQ), and magnetic quadrupole moment (MQ), is calculated as shown in the following [25,26]:

(1) $$p=\int P(r^{\prime })dr^{\prime }$$

(2) $$m=-\frac{i\omega }{2}\int \left[r^{\prime }\times P(r^{\prime })\right]dr^{\prime }$$

(3) $$T=\frac{i\omega }{10}\int \left[(2{r^{\prime }}^{2}P(r^{\prime })-(r^{\prime }\cdot P(r^{\prime }))r^{\prime }\right]dr^{\prime }$$

(4) $$Q^{e}=3\int \left[r^{\prime }P(r^{\prime })+P(r^{\prime })r^{\prime }\right]dr^{\prime }$$

(5) $$Q^{m}=-\frac{2i\omega }{3}\int [r^{\prime }\times P(r^{\prime })]r^{\prime }dr^{\prime }$$

where $P(r^{\prime })$ stands for the polarization induced in the scatter by an incident light wave and $r^{\prime }$ represents the radius-vector of a volume element inside the scattering medium. The total scattering cross section of the higher modes can be expressed as [27]:

(6) I=14πε0[2ω43c3|P|2+2ω43c3|M|2+4ω53c4Im(P⋅T∗)+2ω63C5|T|2+ω620c5|Qe|2+ω620c5|Qm|2]

(7) $$C_{sca}=\frac{I}{I_{inc}}$$

where I is the radiation power from these different multipole moments and $I_{inc}$ is the radiation power of the incident light wave.

The formula to calculate the strength enhancement is:

(8) $$NI={\left(E/E_{0}\right)}^{2}={\left(\sqrt{E_{ex}^{2}+E_{ey}^{2}+E_{ez}^{2}}/E_{0}\right)}^{2}$$

where *E*_0_ is the incident electric field intensity *E*_0_ = 1 V/m).

**Figure 1:**
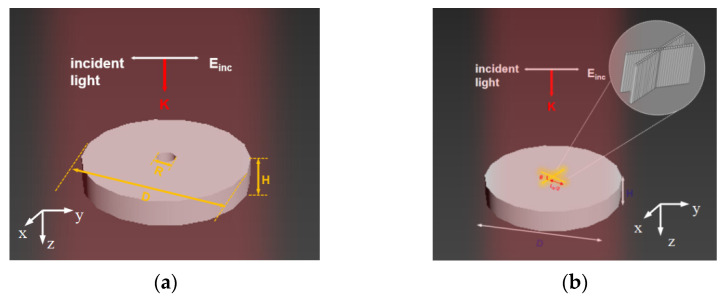
(**a**) Schematic diagram of the GaP nanodisk with a hole, irradiated with y-polarized light incident, and *R* is the diameter of the hole in the disk. (**b**) Extension of the cylindrical hole to form a structure with a fork-shaped bandgap antenna where *D* and *H* are the diameter and height of the disk antenna. Light is limited to the volume of the groove, thus producing powerful local hot spots.

The finite element method (FEM) is implemented to discretize the multipole moment differential equation parametrically. The electromagnetic characteristics of the circular GaP disk with a hole are derived, and the excitation of the primary nonradiative dipole mode is studied. Figure 1a depicts a schematic diagram of the GaP disk irradiated with y-polarized light. In the beginning, the diameter and height of the disk are *D* = 700 nm and *H* = 100 nm, respectively, and the diameter of the hole in the disk, *R*, is 10 nm. A plane wave that is linearly polarized in the y direction impinges vertically along the *z*-axis. The dielectric constant of GaP is extracted from the experimental data of Aspnes and Studna and the background medium is air with a refractive index *n* = 1 [28].

## 3. Results and Discussion

**Figure 2:**
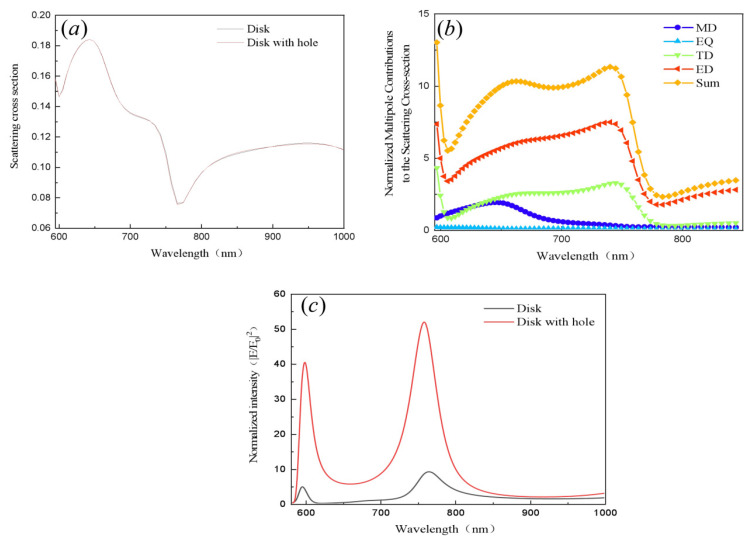
(**a**) Far-field scattering of the disk antenna (black solid line) and the disk with the hole antenna (red solid line). (**b**) Normalized multimode contributions to the scattering cross section of the disk with the hole antenna, where E is the electric field intensity at the detection point and E_0_ is the intensity of the incident electric field. (**c**) Near-field enhancement of the disk antenna (black solid line) and disk with the hole antenna (red solid line).

To investigate the scattering features of different nanoantennas, a comparison of the scattering cross sections of the disk and the slit disk is shown in Figure 2a. Note that the scattering spectrum is almost unchanged. The two obvious tilts around 600 nm and 760 nm reveal the first-order and second-order excited anapole modes, namely, AM1 and AM2. To explicitly reveal the contribution of different anapole modes governed by the calculation of the disk with a hole antenna, the Cartesian multipolar contribution to scattering is shown in Figure 2b. ED and TD contribute significantly to the scattering cross section, which is an electric dipole state [29]. It is clear that the first-order anapole mode at 600 nm originates from the ED, whereas the second-order anapole mode at 760 nm is ascribed to both the ED and TD resonances. Here, the anapole mode is defined as the scattering dark state of the main electric dipole response. This phenomenon suppresses the total radiation energy because of the superposition of mutually canceling internal mode components in the far field [30]. Figure 2c exhibits the intensity enhancement curves of the circular antenna (black solid line) and the disk with a hole antenna (red solid line). The two significant peaks agree with the position of the wave trough in the scattering spectrum. Compared with the near-field enhancement of the disk antenna, the disk with the hole antenna shows an enhancement of greater than five times.

**Figure 3:**
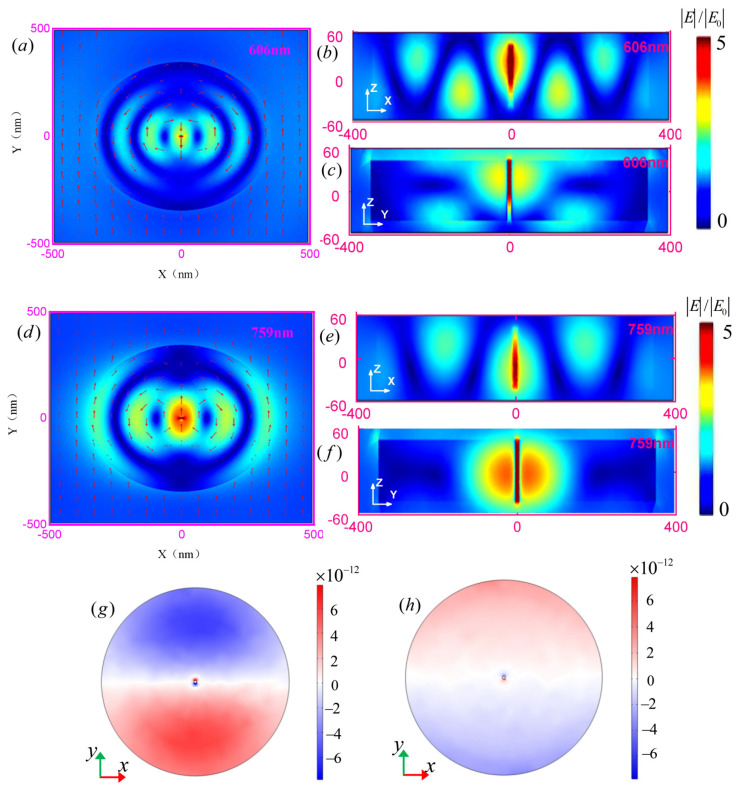
Electric field and surface charge distributions of the GaP disk antenna and the disk with the hole antenna at incident wavelengths of λ = 606 nm (**a**–**c**,**g**) and λ = 759 nm (**d**–**f**,**h**) (Scale bar is 100 nm).

To gain further insight into the electric field enhancement, Figure 3 shows the electric field and surface charge distributions of the gap in the disk at the peak of the voltage enhancement curve (λ = 606 nm and λ = 759 nm). Figure 3a–c shows that the electric field is enhanced in the GaP gap, and that the circular displacement currents on both sides of the slit are different. In contrast to the conduction current in free-electron metal, the displacement current in dielectrics is caused by bound electron oscillation as a result of field penetration and phase delay effects in the dielectric particles [31,32]. Figure 3d–f shows the markedly strengthened field and Figure 3g,h shows the surface charge distributions at incident wavelengths λ of 606 nm and 759 nm. The induced charges are distributed on the surface of the perforated disk, and red and blue indicate positive and negative charges, respectively. The positive and negative charges are distributed on both sides of the disk, with an obvious boundary. The charge density distribution indicates that the peaks at the AM1 and AM2 modes correspond to the dipolar resonance mode, which is considered a dipolar–dipolar mode.

**Figure 4:**
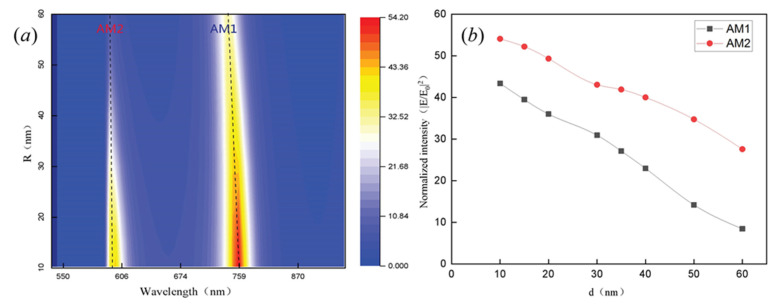
Electric field intensity enhancement of the GaP disk with various gap widths, *d*. The size of the disk is the same as that in Figure 2a. (**a**) Changes in wavelength and inter-slit width, R, affecting the intensity of the electric field. (**b**) Near-field enhancement of the electric field with different gap widths of the disk hole in the AM1 and AM2 modes.

Figure 4 presents the relationship between the electric field enhancement factors and the finite size of the circular hole slit. The size of the disk is the same as that in Figure 2a. Two prominent peaks are shown in the scattering spectrum with enhancement factors larger than 40 and 50, respectively, corresponding with the positions of the two anapole modes. As shown in Figure 4a, different anapole modes move slightly when the inter-slit width of the nanodisk increases from 10 nm to 60 nm. The field strength increases gradually with decreasing circular hole diameters because the circular hole interacts with the enhancement field in the anapole mode, giving rise to larger field strength. Figure 4b shows that the field distribution is stronger in the AM2 mode than AM1 mode because the electric field in the AM2 mode is fixed to a greater extent in the center of the disk.

**Figure 5:**
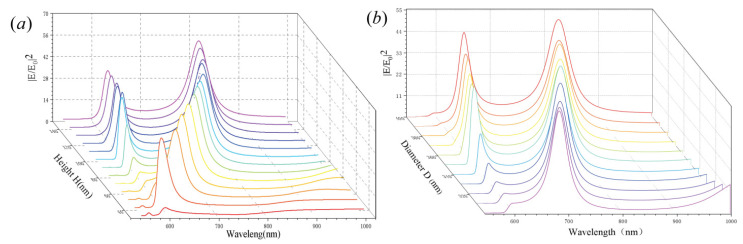
Size-dependent near-field intensity enhancement of the disk with a hole: (**a**) effects of the disk height, H, on the intensity enhancement spectra and (**b**) effects of the disk diameter, *D*, on the intensity enhancement spectra.

Figure 5 shows the near-field intensity enhancement of the disk with a hole as a function of height, *H*, with different diameter, *D*. It is found that the anapole modes exhibit significant redshift with increasing height or diameter. The resonance wavelength of the anapole modes does not change for *D* = 700 nm and *H* = 100 nm, while the electric field intensity shows a small redshift with increasing *H* or *D*. The results clearly illustrate that the size of the disk impacts the near-field intensity enhancement. However, if the size is too large, there is little impact on the disk system.

**Figure 6:**
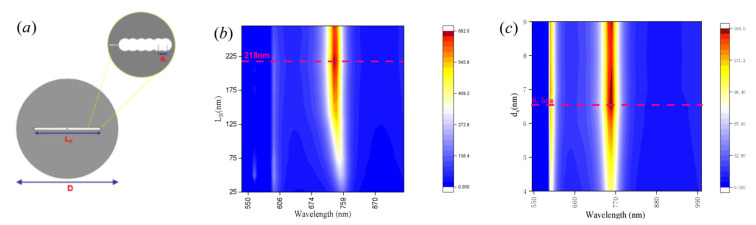
(**a**) Schematic illustration of the GaP disk with a gourd-shaped gap formed by arranging spaced circular holes along the *y*-axis. (**b**,**c**) Electric field enhancement as a function of the spacing between holes, d_s_, or length of the holes and wavelength.

More circular holes are produced on both sides of the initial circular hole and the distance between them is set to be along the *y*-axis, as shown in Figure 6a. Figure 6b,c shows the impact of the distance, *d_s_*, or combined length of holes, *L_B_*, on the electric field enhancement and resonance wavelength. The introduction of circular holes leads to a larger field intensity. It can be seen that the intensity enhancement can correspondingly reach its maximum at 683 nm when the combined length of holes, *L_B_*, is increased from 25 nm to 300 nm. The distance between circular holes shows an optimum value, and when the slit size is not optimal, the interaction with the field is insufficient. After determining the optimal hole pitch, a circular hole of the same size is added to the initial circular hole along the *y*-axis, with the same hole pitch until the electric field reaches a maximum. The resonance wavelength of the anapole mode shows blueshift with increasing gap length because of the smaller disk volume. To clarify the influence of slits with different shapes on the near-field enhancement, we compare the simulation results of near-field intensity enhancement by varying length and width, d_s_, and the results are plotted in Figure S1. As shown in Figure S1, the GaP disk with a gourd-shaped gap formed by arranging spaced circular holes produces strong electric fields with intensity enhancements in comparison to the GaP disk with a slot when increasing the length, *L_s_*, and width, *d_s_*. Therefore, the GaP disk with a gourd-shaped gap formed by arranging spaced circular holes provides a strongly enhanced electric field with the optimal design configuration. In experimental work, the roughness, shape and size of the hole will have some variability. A systematic simulation is performed by varying these three parameters, as shown in Figure S2. The results clearly illustrate that the parameters of the hole have definite effects on the near-field intensity, and the shape and size of the hole have a major impact on the near-field intensity enhancement.

**Figure 7:**
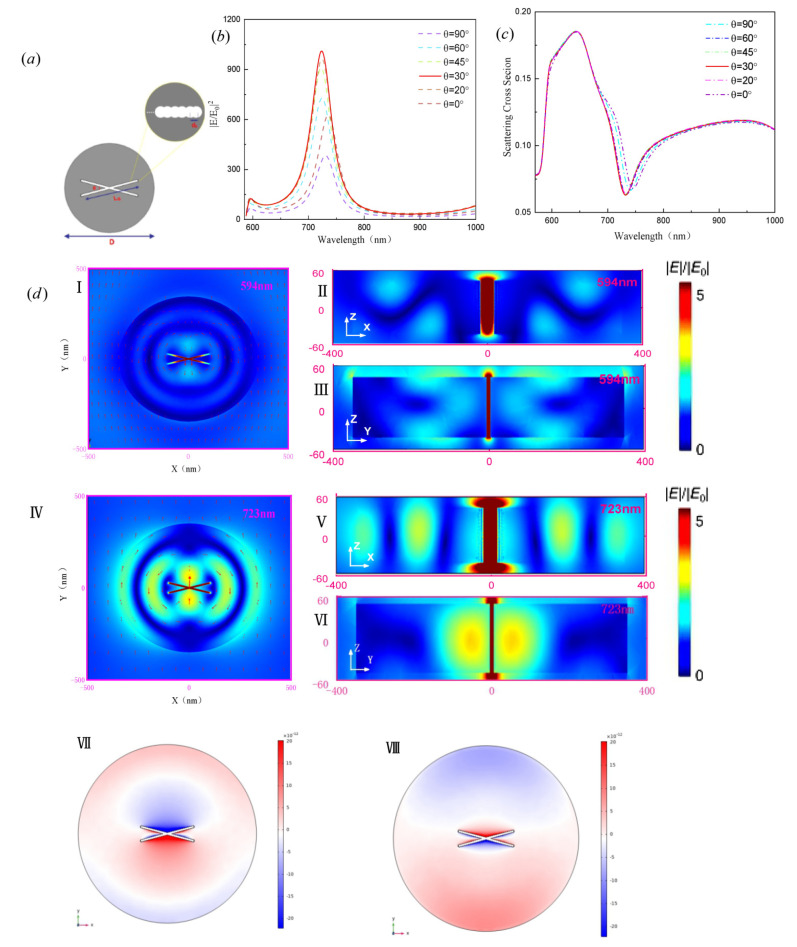
(**a**) Schematic illustration of the nanoantenna with the forked slit. Two cucurbit circular holes with the same combined length of 218 nm are rotated along the *y*-axis to form a new nanoantenna with a fork-shaped slot, where θ is the angle between the two gourd-shaped gaps. (**b**,**c**) Changes in the angle contribution for far-field scattering and near-field enhancement of the nanoantenna with the forked gap. (**d**) Electric field and surface charge distributions of the GaP nanoantenna with the forked gap at incident wavelengths of λ = 594 nm (Ⅰ–Ⅲ, Ⅶ) and λ = 723 nm (Ⅳ–Ⅵ, Ⅷ).

To maximize the electric field, two slits with the optimal length are introduced to the middle of the disk and rotated along the *y*-axis to form a forked slot as shown in Figure 7a. Note that the spectral dependence of the scattering cross section and near-field enhancement as a function of the angle between the two gourd-shaped gaps is observed in Figure 7b,c. There are two obvious peaks in the field intensity enhancement curve with enhancement factors over 100 and 1000, respectively, which are the same as the two anapole modes in the scattering spectrum. The nanoantenna with the forked slit can produce strong electric fields with intensity enhancements exceeding the conventional nanodisk by 100 times, largely outperforming many plasmonic nanostructures with the same gap size (as shown in Figure S3) [33,34]. The large field enhancement caused by the gap can be explained by the boundary condition of the standard component of the displacement. Figure 7d shows the electric field and charge distributions of the GaP forked gap at incident wavelengths of 594 nm and 723 nm. These profiles are characterized by the anapole state, where almost all the field amplitudes circulate in the plane of the disk and most of the energy is confined in the fork-type slot. The dipole mode of the nanoantenna with the forked slit indeed stems from destructive interference of the electric dipole with the circulating dipole. The electric field enhancement is larger than that of the single circular hole slit, resulting in a strong hot spot at the forked slit. For the same reason, a large current density is generated. The field enhancement factors are about 13 and 33.6 in the AM1 and AM2 modes, respectively. Although the electric field in the AM2 mode is bigger, the overall near-field distributions of the two modes are comparable. When the wavelengths are 594 nm and 723 nm, the charge distributions exhibit a dipole distribution due to the opening of the forked slit. Because of the strong electric field, high densities of positive and negative charges are concentrated on both sides of the gap. When replacing the GaP disk with a forked circular hole slit to a GaP disk with a forked slot slit, Figure S4 shows that the intensity enhancement of the GaP disk with holes reaches the maximum as compared to the GaP disk with the slot, illustrating the optimality of the structure.

## 4. Conclusions

A high-performance all-dielectric nanostructure is designed to enhance the multi-resonant electric field. Starting with a conventional disk and opening a circular hole or multiple holes, the field strength increases by a factor of nearly 100 at the maximum value of the electric field generated by the anapole mode. In-depth analysis of the fundamental optical response of the system is carried out by the multipole decomposition method. The spectral evolution and intensity enhancement influenced by geometrical parameters and mode interactions are studied. Analysis of the two factors influencing the spectral evolution and intensity enhancement (interactions between the geometrical parameters and modes) shows results similar to those obtained from metallic plasma systems. Therefore, this novel system can be extended to other materials and frequency ranges. The high field intensity of the GaP nanoantenna with a forked slit suggests a new strategy to obtain single-photon emission in resonant systems.

## Data Availability

The data presented in this study are available upon request from the corresponding author.

## Supplementary Materials

The following are available online at https://www.mdpi.com/article/10.3390/nano11061490/s1, Figure S1: (a) Effects of the length of the slot *L_s_* on the intensity enhancement spectra (b) Effects of the width on the intensity enhancement spectra. Figure S2: The electric field enhancement spectra for nanodisk with different (a) roughness, (b) shape, and (c) size. Figure S3: Comparison of the electric field enhancement of the nanodisk and the forked slit nanodisk. Figure S4: Near-field enhancement of the forked circular hole slit and forked slot slit (a) Effects of the length of the forked slot *L_s_* (b) Effects of the width of the forked slot *R*.
