# Supplementary material for: Optical Anapole Modes in Gallium Phosphide Nanodisk with Forked Slits for Electric Field Enhancement
Source: Nanomaterials (Basel). 2021 Jun 4;11(6):1490. doi: 10.3390/nano11061490 (PMC8229694; doi:10.3390/nano11061490)
Supplement: Supplementary file 1 [file nanomaterials-11-01490-s001.zip › nanomaterials-1217496-supplementary.pdf]

nanomaterials

MDPI

Article

Optical Anapole Modes in Gallium Phosphide Nanodisk with Forked Slits for Electric Field Enhancement

Jingwei Lv, He Zhang, Chao Liu*, Zao Yi*, Famei Wang, Haiwei Mu, Xianli Li, Tao Sun and Paul K. Chu*

To clarify the influence of slits with different shapes on the near-field enhancement, we compare the simulation results of near-field intensity enhancement by varying length and width *d*, and the results are plotted in Figure S1. In this case, the network of holes was simply replaced by a slot of length L_s and width d_s. As shown in Figure S1, the GaP disk with a gourd-shaped gap formed by arranging spaced circular holes produces strong electric fields with intensity enhancements in comparison to the GaP disk with a slot when increasing the length L_s and width d_s. Therefore, the GaP disk with a gourd-shaped gap formed by arranging spaced circular holes provides a strongly enhanced electric field with the optimal design configuration.

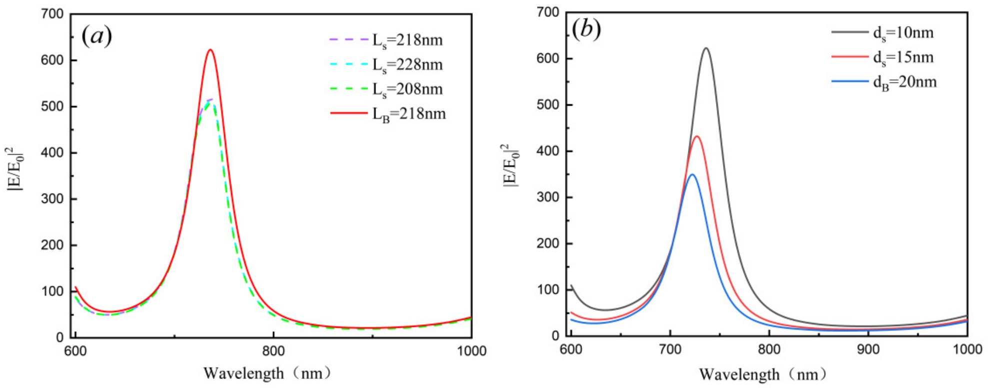

Figure S1. (**a**) Effects of the length of the slot L_s on the intensity enhancement spectra; (**b**) effects of the width d_s on the intensity enhancement spectra.

In an experimental work, the roughness, shape and size of the hole will have some variability. A systematic simulation is performed by varying three parameters, the size, shape and roughness, as shown in Figure S2. During the manufacturing process, the holes have some variability due to the unavoidable reasons that make the structure rough. The comparison of the near-field enhancement of antennas with different roughness is shown in Figure S2a. Note that several rough holes in the nanodisk change the near-field enhancement of the antenna only slightly. When the shape is changed from a cylinder to a square, the shape of the holes will become a simple rectangle; the comparison of near-field enhancement is shown in Figure S2b. It can be seen that the effect of the slit composed of a cylinder shows a higher field intensity than that of the slot. As shown in Figure S2c, different peaks move forward to the shorter wavelength when the size of the hole radius d increases from 10 nm to 20 nm. The results clearly illustrate that the size of the hole impacts the near-field intensity enhancement.

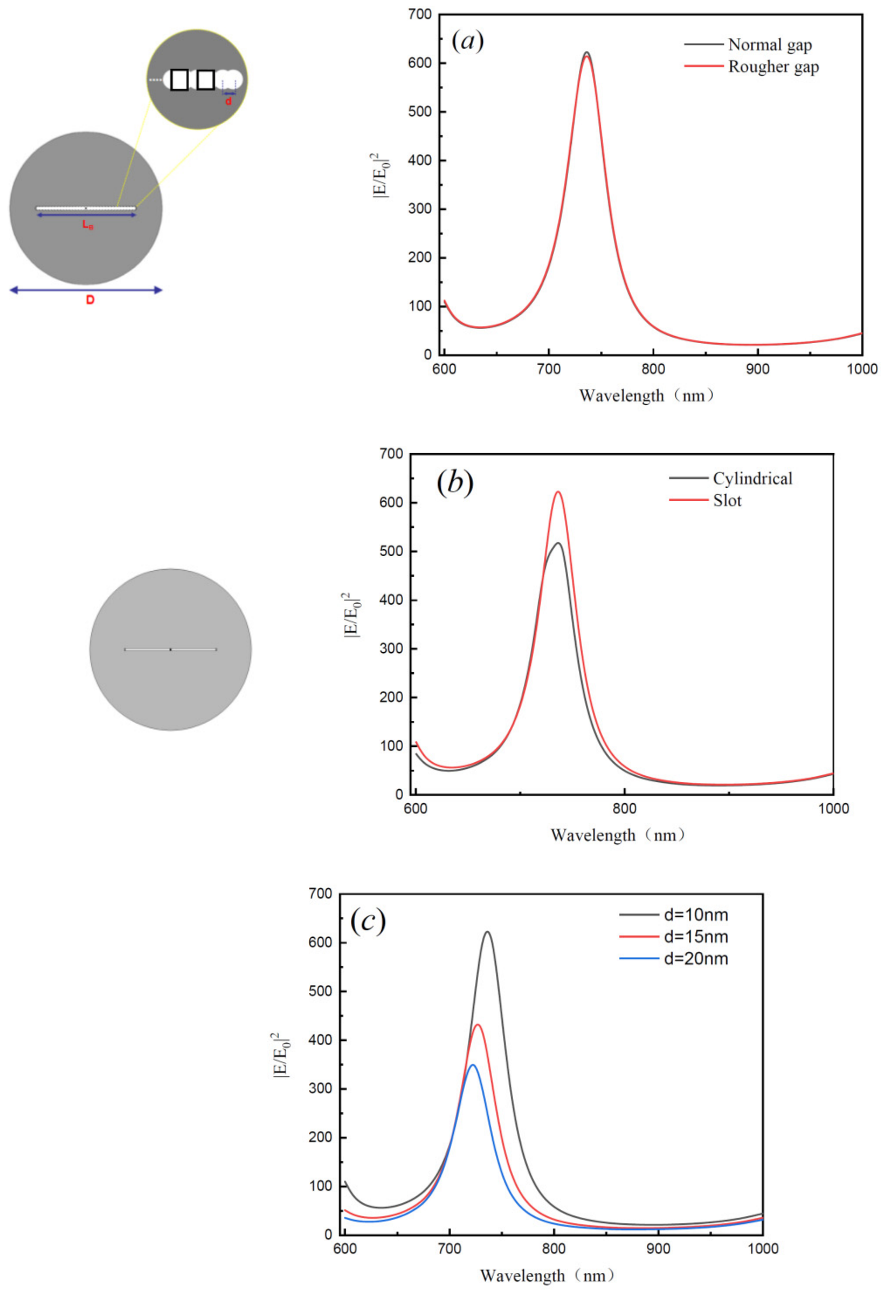

Figure S2. The electric field enhancement spectra for nanodisks with different (a) roughness, (b) shape, and (c) size.

We extract the maximum data on the electric field enhancement of the nanodisk and the forked slit nanodisk from Figure 2c and Figure 7b and redraw the comparison graph in Figure S4. The nanoantenna with the forked slit can produce strong electric fields with intensity enhancements more than 100 times those of the conventional nanodisk, largely outperforming many plasmonic nanostructures with the same gap size.

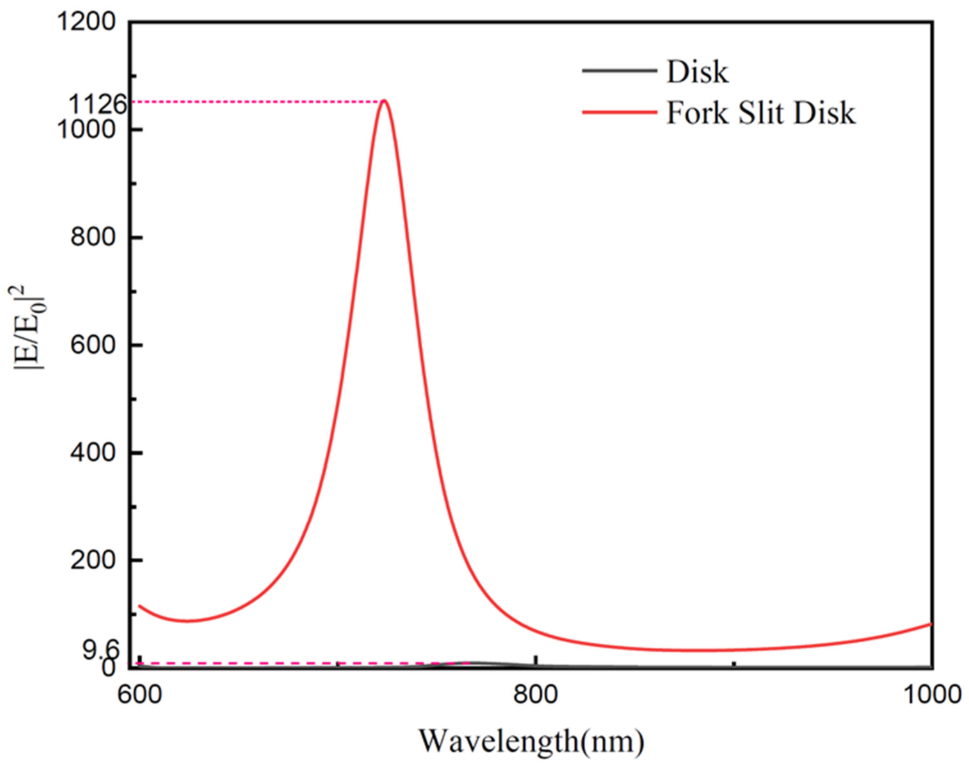

Figure S3. Comparison of the electric field enhancement of the nanodisk and the forked slit nanodisk.

By simply replacing the GaP disk with a forked circular hole slit with a GaP disk with a forked slot slit, Figure S4 shows that the intensity enhancement of the GaP disk with holes reaches the maximum when compared with GaP disk with a slot by increasing the length and width, exhibiting the optimality of structure.

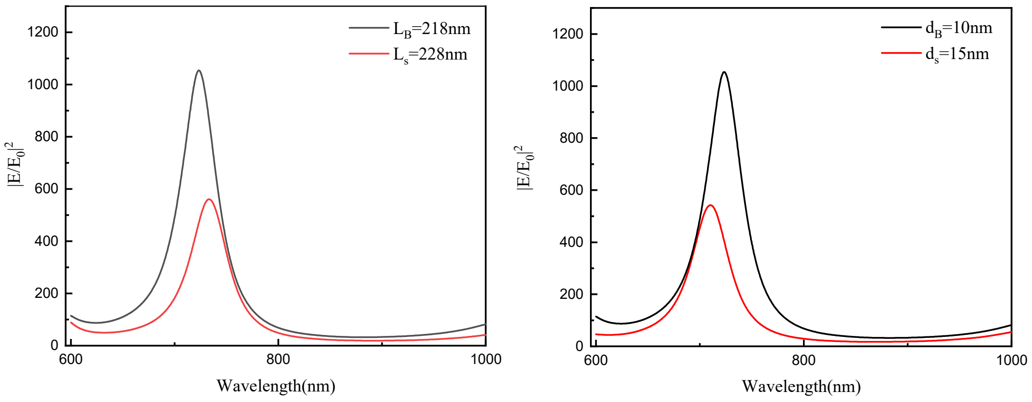

Figure S4. Near-field enhancement of the forked circular hole slit and forked slot slit:. (**a**) effects of the length of the forked slot L_s; (**b**) effects of the width of the forked slot *R*.
